# Reply to Bae, I.-S. Comment on “Lee et al. Conservative Treatment with Teriparatide Versus Vertebroplasty for Acute Osteoporotic Vertebral Compression Fractures: A Meta-Analysis. *J. Clin. Med.* 2025, *14*, 3967”

**DOI:** 10.3390/jcm15124697

**Published:** 2026-06-17

**Authors:** Subum Lee, Junseok W. Hur, Younggyu Oh, Sungjae An, Yeongu Chung, Danbi Park, Jin Hoon Park

**Affiliations:** 1 Department of Neurosurgery, Korea University Anam Hospital, Korea University College of Medicine, Seoul 02841, Republic of Korea; lee_s@korea.ac.kr (S.L.);; 2 Department of Neurosurgery, Kangbuk Samsung Hospital, Sungkyunkwan University School of Medicine, Seoul 03181, Republic of Korea; 3 Department of Neurological Surgery, Asan Medical Center, University of Ulsan College of Medicine, 88 Olympic-ro 43-gil, Songpa-gu, Seoul 05505, Republic of Korea

We thank Dr. Bae for the thoughtful commentary [1] on our meta-analysis [2], and respond to each point below.

## 1. Statistical Power and Small Sample Size

We agree that including only five studies (n = 326) limits statistical power, reflecting the current paucity of head-to-head TP vs. VP comparisons. However, an indirect but compelling line of evidence supports the directional consistency of our findings. Large-scale RCTs and Cochrane reviews have consistently failed to demonstrate a significant clinical benefit of VP over conventional conservative treatment [3,4,5]. Separately, a recent network meta-analysis ranked TP as the most effective conservative intervention for pain relief and quality of life in OVCFs [6]. Taken together, if VP is not superior to conventional conservative care, and TP is the best-performing conservative strategy, it is plausible that TP may be superior to VP—a premise our meta-analysis directly tests and supports. Large-scale multicenter RCTs remain essential to confirm this conclusion, as Dr. Bae recommends.

## 2. Heterogeneity in VAS Scores

We acknowledge the high heterogeneity (I^2^ > 95%) in VAS outcomes as a genuine limitation. However, heterogeneity was substantially reduced in age-based subgroups, to 64.10% in patients < 75 years at 12 months (pooled MD = −2.01, 95% CI −2.20 to −1.82, favoring TP). In the ≥75 years subgroup at 6 months, only a single study (Su et al. [7]) was available (MD = −1.96, 95% CI −2.15 to −1.77), precluding I^2^ calculation but demonstrating a consistent and directionally strong treatment effect favoring TP. These findings suggest that patient age is a clinically meaningful effect modifier. Furthermore, the only study showing a directional advantage for VP (Jung et al. [8]) used denosumab co-administered with TP, introducing a likely independent confounding factor. Sensitivity analysis excluding this study increased both the effect size and homogeneity favoring TP. Importantly, despite the statistical heterogeneity, the direction of treatment effect consistently favored TP at 6 and 12 months across all studies, lending qualitative support to our conclusions.

## 3. Exclusion of Kyphoplasty (KP)

Although balloon KP is one of the most widely performed augmentation procedures for OVCFs and carries substantial clinical importance, it was excluded to preserve procedural homogeneity, as KP and VP differ substantially in balloon inflation, cement volume, and biomechanical technique. This was a deliberate methodological decision rather than a reflection of KP’s clinical relevance, and we acknowledge that it limits generalizability. Recent data—including 12-month TP after KP [9], a comparative analysis of anti-osteoporosis medications after balloon KP [10], and a 2025 prospective multicenter study of osteoanabolic therapy after balloon KP [11]—provide promising evidence. Future meta-analyses should incorporate KP with intervention-level subgroup stratification.

## 4. Cost-Effectiveness and Patient Compliance

These practical concerns are valid and clinically important. Daily injection burden and the 24-month lifetime cap are real barriers; a recent study reported only 54% of patients completing the full TP course [12]. However, biosimilar TP has demonstrated fracture-prevention efficacy equivalent to the reference product (adjusted HR 0.95, 95% CI 0.82–1.11) at approximately 34% lower monthly cost [13], directly reducing the economic barrier that contributes to the 46% non-completion rate, while less-frequent formulations may further improve adherence by reducing injection burden [14]. Future studies should formally evaluate the cost-effectiveness of TP in the acute OVCF setting, as this remains an important unresolved question for clinical decision-making.

## 5. Radiologic Progression and Vertebral Collapse

Dr. Bae correctly references Chen et al. [15], who reported greater vertebral collapse with TP versus VP. Importantly, that study used only 3-month TP with 6-month follow-up, whereas studies in our meta-analysis used regimens of 12–18 months. Short-term height loss with TP relative to VP is mechanistically expected given VP’s immediate mechanical support; it does not reflect long-term structural outcomes. Indeed, Yang et al. demonstrated that TP was associated with fewer refractures and better preservation of vertebral height of cemented vertebrae over one year [16]. Our analysis showed no statistically significant difference in kyphotic angle or anterior height loss at 12 months, and TP’s advantage in preventing new-onset OVCFs (OR 0.15, 95% CI 0.04–0.51) remains a critical long-term benefit [2].

## 6. Conclusions

We are grateful to Dr. Bae for the insightful critique. The methodological limitations of our study are real and candidly acknowledged. Recent literature reinforces the long-term clinical value of TP in acute OVCFs while also clarifying the patient profiles in which benefits are most consistent. In particular, the decline in statistical heterogeneity—from I^2^ >95% overall to 64.1% in patients aged <75 years at 12 months, with the ≥75 years subgroup at 6 months represented by a single consistent study strongly favoring TP (MD = −1.96, 95% CI −2.15 to −1.77)—suggests that the benefit of TP is most consistent in identifiable patient subgroups, particularly the elderly population most frequently affected by acute OVCFs. We maintain that TP represents a meaningful alternative to cement augmentation, particularly for patients at high risk of adjacent-level fractures, and we agree that adequately powered RCTs with standardized protocols—including both VP and KP comparators—are the necessary next step.
